# Teofilina para o Alívio da Dispneia Relacionada ao Ticagrelor

**DOI:** 10.36660/abc.20201076

**Published:** 2021-07-15

**Authors:** Marcelo Sanmartin-Fernandez, Jose Luis Zamorano

**Affiliations:** 1 Hospital Universitario Ramon y Cajal Madri Espanha Hospital Universitario Ramon y Cajal - Serviço de Cardiologia,Madri - Espanha

**Keywords:** Ticagrelor, Teofilina, Síndrome Coronariana Aguda

## Introdução

A dispneia é uma das reações mais comuns ao ticagrelor, levando à sua descontinuação prematura em 15 a 20% dos pacientes.^1^ O mecanismo não é claro, mas acredita-se que esteja relacionado ao aumento da concentração de adenosina no tecido ou à inibição reversiva e direta dos receptores P2Y_12 _nos neurônios sensoriais.^2^

A teofilina tem sido usada como broncodilatador desde 1922, e seus efeitos aparentam ser parcialmente mediados por meio do bloqueio não-seletivo dos receptores de adenosina.^3^

A hipótese dos autores é a de que a teofilina poderia atenuar a dispneia causada pela administração do ticagrelor, e reportaram sua experiência clínica inicial com 10 pacientes.

## Métodos

Os critérios de inclusão foram: 1) presença de dispneia moderada ou grave após pelo menos uma dose de ticagrelor, não explicada por causas cardíacas ou pulmonares; 2) Acompanhamento em uma unidade de terapia intensiva do coração; 3) Concordância formal do paciente pelo uso compassivo da teofilina.

Uma única dose de 200 mg de teofilina foi administrada por infusão salina, diluída em 100ml-0,9%, após 20 minutos. Todos os pacientes fizeram um exame clínico completo, eletrocardiograma e ecocardiograma antes da administração do medicamento, além do monitoramento contínuo da saturação do O_2_, eletrocardiograma e medição de pressão arterial não-invasiva. O eletrocardiograma e o ecocardiograma foram realizados à beira do leito, com a única intenção de excluir causas alternativas da dispneia, como isquemia continua, insuficiência cardíaca ou broncoespasmo.

A avaliação dos sintomas e o exame físico foram realizados antes e imediatamente após a infusão. Cada paciente forneceu autorização específica e verbal para o uso compassivo da teofilina nesta situação. Todos os pacientes deram consentimento para publicação de dados anônimos.

## Resultados

De janeiro de 2017 a dezembro de 2019, 1.437 pacientes foram internados no hospital com síndromes coronarianas agudas, dos quais 29 (3,1%) apresentaram dispneia moderada a grave durante a hospitalização, presumidamente relacionada à administração do ticagrelor. Um total de 10 pacientes foram tratados empiricamente com 200 mg de teofilina intravenosa, já que seus sintomas foram considerados suficientemente consideráveis para requerer manobras terapêuticas ( Tabela 1 ). A idade mediana foi 75 anos (intervalo interquartil 61-80 anos), seis pacientes tiveram infartos do miocárdio prévios, cinco tinham diabetes mellitus tipo 2, quatro eram fumantes e nenhum tinha doença pulmonar obstrutiva crônica prévia. Em seis pacientes, os sintomas começaram após a administração oral de uma dose de 180 mg em bolus, incluindo dois pacientes que já tinham recebido o clopidogrel.

**Tabela 1:** – Características dos pacientes. Todos os pacientes receberam ticagrelor como agente antiplaquetário inicial, com uma dose oral de 180 mg em bolus, exceto os pacientes #8 e #9, que receberam clopidogrel antes e depois mudaram para o ticagrelor 90 mg bid.

Paciente	Idade (anos)	Peso (kg)	Sexo	Síndrome coronariana aguda	Saturação O_2_ de base (%)	Inibidor P2Y_12_ inicial	Início da dispneia (horas depos da dose de 180 mg em bolus)	Amenização da dispneia	Liberação do inibidor P2Y_12_
1	56	75	Masculino	STEMI	98	Ticagrelor	48	Completa	Prasugrel
2	80	54	Masculino	STEMI	99	Ticagrelor	36	Parcial	Clopidogrel
3	76	80	Feminino	STEMI	93	Ticagrelor	2	Completa	Clopidogrel
4	57	83	Masculino	STEMI	99	Ticagrelor	40	Completa	Prasugrel
5	78	68	Masculino	NSTEMI	97	Ticagrelor	2	Completa	Clopidogrel
6	48	85	Masculino	STEMI	95	Ticagrelor	12	Parcial	Clopidogrel
7	71	100	Masculino	NSTEMI	97	Ticagrelor	2	Completa	Prasugrel
8	82	75	Masculino	STEMI	95	Clopidogrel	2	Completa	Clopidogrel
9	73	68	Feminino	NSTEMI	98	Clopidogrel	3	Completa	Clopidogrel
10	83	72	Masculino	NSTEMI	97	Ticagrelor	1	Completa	Clopidogrel

Após a administração da teofilina, todos os pacientes reportaram a melhora na falta de ar, embora dois pacientes tenham reportado somente um alívio parcial. Em um dos pacientes com resposta parcial, uma segunda dose de 200 mg em bolus foi administrada uma hora depois, com a completa resolução dos sintomas. Todos os pacientes foram tratados com um diferente inibidor P2Y_12 _após o evento. Um paciente (paciente #3) recebeu tratamento de teofilina de 200-mg bid, oralmente, junto com o ticagrelor, antes de os autores decidirem mudar para o clopidogrel previamente à alta. Nenhum paciente apresentou trombose de stent ou outros eventos trombóticos durante a hospitalização. Um paciente de 82 anos, com infarto do miocárdio sem elevação do ST, tratado de forma conservadora e com alta após o clopidogrel, foi readmitido depois de 20 dias com outro infarto do miocárdio e choque cardiogênico, e morreu apesar da revascularização ter sido bem-sucedida.

## Discussão

Pela primeira vez, os efeitos favoráveis da teofilina são reportados na resolução da dispneia relacionada ao uso de ticagrelor em pacientes com síndromes coronarianas agudas. Embora esta experiência clínica inicial tenha sido muito positiva, esses achados devem ser considerados como exploratórios devido à falta de um grupo controle.

**Figura 1:**
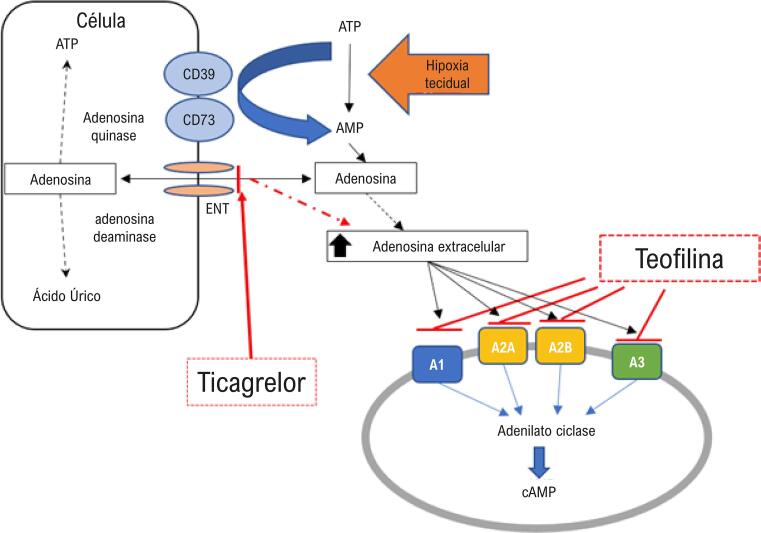
- Lesão do tecido e hipóxia estimulam a formação de adenosina no espaço extracelular pela degradação da adenosina trifosfato (ATP) e da adenosina difosfato (ADP) por meio da ação de nucleotidases como CD39 e CD73. A rápida degradação da adenosina intracelular ocorre pela adenosina quinase e pela adenosina deaminase, depois de serem transportadas com os transportadores de nucleosídeos independentes de sódio (ENT). Como o ticagrelor inibe a membrana dos ENT, a adenosina se acumula no espaço extracelular, levando a efeitos mediados pela adenosina, como a estimulação dos neuroreceptores no pulmão, provavelmente ao se ligarem aos receptores da adenosina (A1, A2A, A2B, e A3), e, mais tarde, a interação com o adenilato ciclase e a formação do monofostafo cíclico de adenosina (cAMP). O bloqueio da teofilina em receptores da adenosina provavelmente explica a inibição dos efeitos mediados pela adenosina.^4^

Embora os mecanismos por trás do alívio da dispneia possam ser multifatoriais, esses achados preliminares fortalecem a hipótese da adenosina para a dispneia causada pela inibição reversiva dos receptores P2Y_12 _( Figura 1 ). Em um estudo duplo-cego, controlado com placebo, Wittfeldt et al.^5^demonstraram que uma dose de 5 mg/kg de teofilina amenizou os efeitos de uma infusão incremental de adenosina em 40 voluntários tratados com 180 mg de ticagrelor na velocidade do fluxo coronário e sintomas de dispneia, medidos pela escala de Borg. Os resultados apresentados estendem esses achados à população de pacientes com síndromes coronarianas agudas, e usando uma dose menor de teofilina.

A teofilina é derivada da xantina, muito usada no tratamento da asma por muitas décadas, embora seus mecanismos moleculares precisos de ação sejam incertos.^3 , 6^ Entre os mecanismos terapêuticos propostos, foi demonstrado que a teofilina promove a inibição não-seletiva da fosfodiesterase, o antagonismo dos receptores da adenosina e seus efeitos anti-inflamatórios, como a liberação da interleucina-10, a prevenção da translocação do fator de transcrição do fator nuclear kappa B (FN-kB), e a ativação das histonas desacetilases, que melhoram o efeito anti-inflamatório dos corticosteroides. Os autores acreditam que a amenização da dispneia induzida pelo ticagrelor se deva ao bloqueio não-seletivo dos receptores de adenosina, prevenindo a formação intracelular de monofosfato cíclico de adenosina ( Figura 1 ) no tecido intersticial do pulmão, considerando que não há broncoespasmo ou atividade inflamatória local neste cenário clínico.

Não houve efeitos adversos da administração da teofilina, mas as doses selecionadas foram menores do que aquelas recomendadas para asma aguda (5 mg/kg). Não há relatos de resposta pró-trombótica à teofilina. Na verdade, alguns estudos sugerem maior inibição de plaquetas com esta droga.^7^

Embora esses achados requeiram confirmação por meio de ensaios clínicos, uma combinação fixa de teofilina e ticagrelor poderia representar um importante passo para evitar frequentes descontinuações do tratamento com ticagrelor.
