# Supplementary material for: Teofilina para o Alívio da Dispneia Relacionada ao Ticagrelor
Source: Arq Bras Cardiol. 2021 Jul 15;117(1):146–8. [Article in Portuguese] doi: 10.36660/abc.20201076 (PMC8294732; doi:10.36660/abc.20201076)
Supplement: Translation

# Translation: Theophylline for Attenuating Ticagrelor-Related Dyspnea

**Authors:** Marcelo Sanmartin-Fernandez, Jose Luis Zamorano

**Affiliations:** 1 Spain Hospital Universitario Ramon y Cajal - Serviço de Cardiologia, Madri - Spain

**Keywords:** Ticagrelor, Theophylline, Acute Coronary Syndrome

## Introduction

Dyspnea is one of the most frequent adverse reaction of ticagrelor, leading to premature discontinuation in 15-20% of the patients.^1^The mechanism is unclear, but it is thought to be related to increased tissue adenosine concentrations or to the direct reversible inhibition of P2Y_12_receptors on sensory neurons.^2^

Theophylline has been used as a bronchodilator since 1922, and its effects seem to be partly mediated through nonselective adenosine receptors blockade.^3^

The authors hypothesized that theophylline could attenuate the dyspnea caused by ticagrelor administration and reported their initial clinical experience with 10 patients.

## Methods

Inclusion criteria were: 1) Presence of moderate or severe dyspnea after at least one dose of ticagrelor, not explained by cardiac or pulmonary causes; 2) Follow-up in a Cardiac Intensive Care unit; 3) Patient’s formal agreement to the compassionate use of theophylline.

A single dose of 200-mg theophylline was administered, diluted in 100 ml-0.9% saline infusion over 20 min. All patients had a thorough clinical exam, electrocardiogram and echocardiogram performed before drug administration, and continuous monitoring of O_2_saturation, electrocardiogram, and noninvasive blood pressure. Electrocardiogram and echocardiograms were performed at bedside, with the sole intention of excluding alternative causes of dyspnea such as ongoing ischemia, heart failure, or bronchospasm.

Assessment of symptoms and physical exam was performed before and immediately after infusion. Each patient provided specific verbal authorization for compassionate use of theophylline in this situation. All patients granted consent for publication of anonymous data.

## Results

From January/2017 to December/2019, 1,437 patients were admitted to the hospital with acute coronary syndromes, of whom 29 (3.1%) had moderate to severe dyspnea during admission presumably related to ticagrelor administration. A total of 10 patients were empirically treated with 200-mg IV theophylline because their symptoms were deemed to be sufficiently important to require therapeutic maneuvers ( Table 1 ). The median age was 75 years (IQ range 61-80 years), six patients had previous myocardial infarctions, five patients had type 2 diabetes mellitus, four patients were smokers, and none had previous pulmonary obstructive chronic disease. In six patients, symptoms begun after orally receiving the 180-mg bolus dose, including two patients with previous administration of clopidogrel.

**Table 1:** – Patients’ characteristics. All patients received ticagrelor as the initial antiplatelet agent with an oral bolus dose of 180 mg, except patients #8 and #9, who received clopidogrel first and then switched to ticagrelor 90 mg bid

Patient	Age (years)	Weight (kg)	Sex	Acute Coronary Syndrome	Baseline O_2_Saturation (%)	Initial P2Y_12_inhibitor	Dyspnea onset (hours after 180 mg bolus dose)	Attenuation of dyspnea	Discharge P2Y_12_inhibitor
1	56	75	Male	STEMI	98	Ticagrelor	48	Complete	Prasugrel
2	80	54	Male	STEMI	99	Ticagrelor	36	Partial	Clopidogrel
3	76	80	Female	STEMI	93	Ticagrelor	2	Complete	Clopidogrel
4	57	83	Male	STEMI	99	Ticagrelor	40	Complete	Prasugrel
5	78	68	Male	NSTEMI	97	Ticagrelor	2	Complete	Clopidogrel
6	48	85	Male	STEMI	95	Ticagrelor	12	Partial	Clopidogrel
7	71	100	Male	NSTEMI	97	Ticagrelor	2	Complete	Prasugrel
8	82	75	Male	STEMI	95	Clopidogrel	2	Complete	Clopidogrel
9	73	68	Female	NSTEMI	98	Clopidogrel	3	Complete	Clopidogrel
10	83	72	Male	NSTEMI	97	Ticagrelor	1	Complete	Clopidogrel

After theophylline administration, all patients reported attenuation of the shortness of breath, although two patients only reported a partial relief. In one of the patients with partial response, a second 200-mg bolus dose was administered one hour later with complete resolution of symptoms. All patients were treated with a different P2Y_12_inhibitor after the event. One patient (patient #3) further received 200-mg bid theophylline treatment, orally, along with ticagrelor, before the authors decided to switch to clopidogrel prior to discharge. No patient presented stent thrombosis or other thrombotic events during admission. An 82-year-old patient, with non-ST elevation myocardial infarction conservatively treated and discharged with clopidogrel, was readmitted after 20 days with a repeated myocardial infarction and cardiogenic shock and died despite successful revascularization.

## Discussion

For the first time, the favorable effects of theophylline are reported in the resolution of ticagrelor-related dyspnea in patients with acute coronary syndromes. Although this initial clinical experience was strikingly positive, these findings should be considered exploratory due to the lack of a control group.

Although mechanisms behind dyspnea alleviation may be multifactorial, these preliminary findings represent a strong case for the adenosine hypothesis of dyspnea caused by reversible P2Y_12_inhibition ( Figure 1 ). In an elegant double-blind, placebo-controlled study, Wittfeldt et al.^5^showed that a 5-mg/kg dose of theophylline attenuated the effects of an incremental adenosine infusion of 40 volunteers treated with 180-mg ticagrelor on coronary blood flow velocity and dyspnea symptoms, measured by the Borg scale. The present results extend these findings to a population of patients with acute coronary syndromes and using a smaller dose of theophylline.

**Figure 1:** Tissue damage and hypoxia stimulates adenosine formation in extracellular space by degradation of adenosine triphosphate (ATP) and adenosine diphosphate (ADP) through the action of nucleotidases such as CD39 and CD73. Rapid degradation of adenosine intracellularly occurs by adenosine kinase and adenosine deaminase, after being taken up through sodium-independent nucleoside transporters (equilibrative nucleoside transporter (ENT)). As ticagrelor inhibits the ENT membrane, adenosine accumulates in the extracellular space, leading to adenosine-mediated effects, such as stimulation of lung neuroreceptors, probably through binding to adenosine membrane receptors (A1, A2A, A2B, and A3), and later to interaction with adenylyl cyclase and formation of cyclic adenosine monophosphate (cAMP). Theophylline blockade of adenosine receptors probably explains inhibition of adenosine-mediated effects.^4^

Theophylline is a xanthine derivative that has been commonly used in the treatment of asthma for several decades, although its precise molecular mechanism of action is uncertain.^3 , 6^Among the proposed therapeutical mechanisms, it has been shown that theophylline promotes nonselective phosphodiesterase inhibition, antagonism of adenosine receptors and anti-inflammatory effects, such as interleukin-10 release, prevention of translocation of nuclear factor kappa B (NF-𝛋B) transcription factor, and activation of histone deacetylases, which enhances the anti-inflammatory effect of corticosteroids. The authors believe that the attenuation of ticagrelor-induced dyspnea occurs through nonselective blockade of adenosine membrane receptors, preventing intracellular formation of cyclic adenosine monophosphate ( Figure 1 ) in the interstitial lung tissue, considering that there is no objective bronchospasm or local inflammatory activity in this clinical setting.

There were no adverse effects from the theophylline administration, but the selected doses were smaller than those recommended for acute asthma (5 mg/kg). There are no reports of a prothrombotic response to theophylline. In fact, some studies suggest enhanced platelet inhibition with this drug.^7^

Although the present findings require confirmation in clinical trials, a fixed combination of theophylline and ticagrelor could represent an important step for avoiding frequent discontinuations of ticagrelor treatment.
